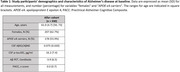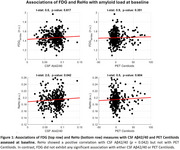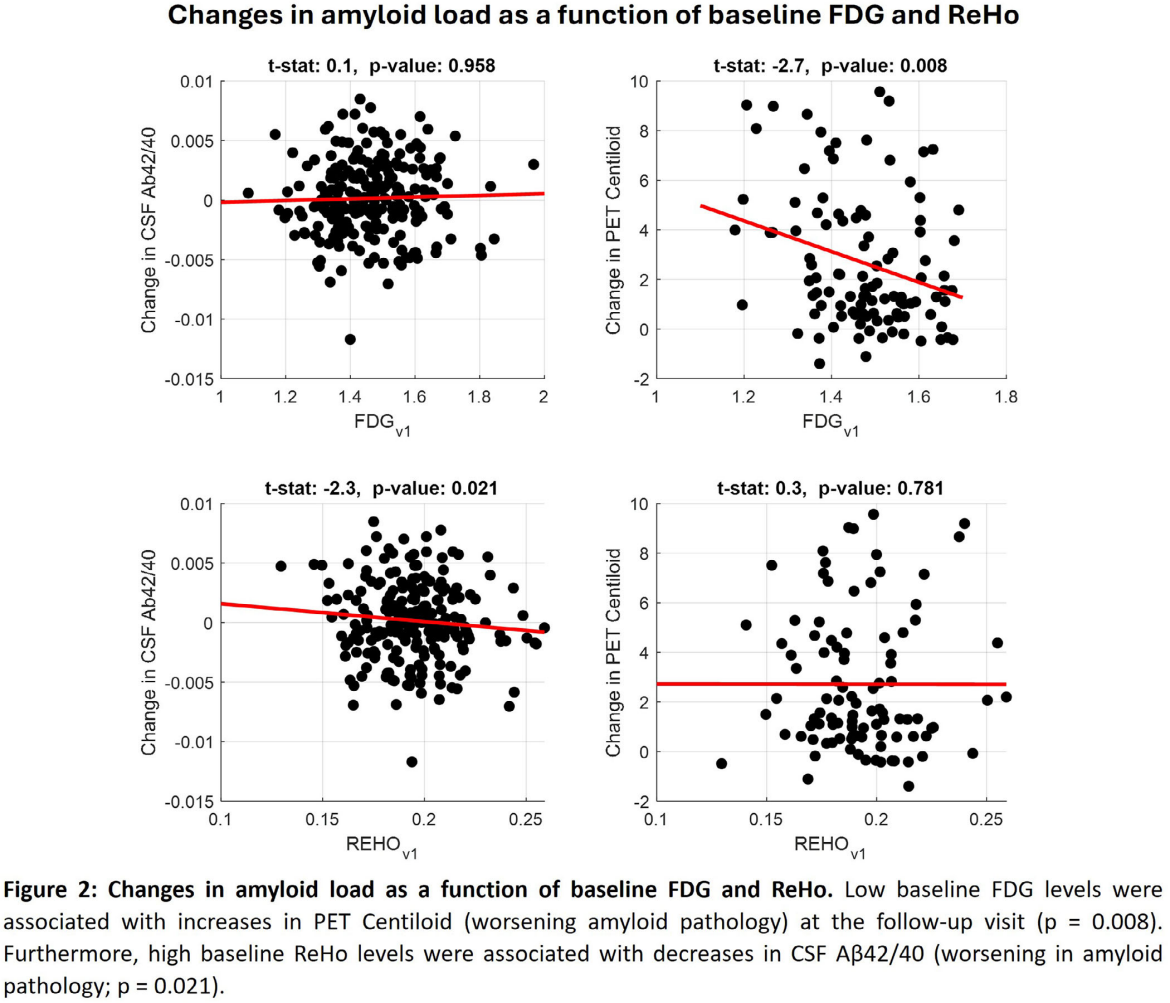# Cerebral glucose metabolism and regional homogeneity as predictors of amyloid burden changes in preclinical AD

**DOI:** 10.1002/alz70856_100911

**Published:** 2025-12-25

**Authors:** Michalis Kassinopoulos, Aldana Lizarraga, Mahnaz Shekari, Jordi Huguet, Marc Suárez‐Calvet, Marta Milá‐Alomá, Kaj Blennow, Henrik Zetterberg, Gwendlyn Kollmorgen, Clara Quijano‐Rubio, Juan Domingo Gispert, Gemma Salvadó, David Vállez‐Garcia, Raffaele Cacciaglia, ALFA study

**Affiliations:** ^1^ Barcelonaβeta Brain Research Center (BBRC), Pasqual Maragall Foundation, Barcelona, Spain; ^2^ BarcelonaBeta Brain Research Center (BBRC), Barcelona, Spain; ^3^ Hospital del Mar Research Institute, Barcelona, Barcelona, Spain; ^4^ Servei de Neurologia, Hospital del Mar, Barcelona, Spain; ^5^ Barcelonaβeta Brain Research Center (BBRC), Barcelona, Spain; ^6^ Department of Psychiatry and Neurochemistry, Institute of Neuroscience and Physiology, University of Gothenburg, Mölndal, Sweden; ^7^ Clinical Neurochemistry Laboratory, Sahlgrenska University Hospital, Mölndal, Sweden; ^8^ Hong Kong Center for Neurodegenerative Diseases, Hong Kong, Science Park, China; ^9^ Department of Neurodegenerative Disease, UCL Queen Square Institute of Neurology, University College London, London, ‐, United Kingdom; ^10^ Clinical Neurochemistry Laboratory, Sahlgrenska University Hospital, Gothenburg, Sweden; ^11^ Institute of Neuroscience and Physiology, University of Gothenburg, Gothenburg, Mölndal, Sweden; ^12^ Wisconsin Alzheimer's Disease Research Center, School of Medicine and Public Health, University of Wisconsin‐Madison, Madison, WI, USA; ^13^ UK Dementia Research Institute at UCL, London, United Kingdom; ^14^ Roche Diagnostics GmbH, Penzberg, Germany; ^15^ Roche Diagnostics International Ltd., Rotkreuz, Switzerland; ^16^ Hospital del Mar Research Institute, Barcelona, Spain; ^17^ Centro de Investigación Biomédica en Red de Bioingeniería, Biomateriales y Nanomedicina (CIBER‐BBN), Madrid, Spain; ^18^ University Pompeu Fabra, Barcelona, Spain; ^19^ Hospital del Mar Research Institute (IMIM), Barcelona, Spain; ^20^ Department of Clinical Sciences, Clinical Memory Research Unit, Lund University, Lund, Spain; ^21^ Department of Radiology and Nuclear Medicine, Amsterdam UMC, Amsterdam, Netherlands; ^22^ Centro de Investigación Biomédica en Red de Fragilidad y Envejecimiento Saludable (CIBERFES), Madrid, Spain

## Abstract

**Background:**

Regional homogeneity (ReHo) is a widely used measure in resting‐state functional magnetic resonance imaging (rs‐fMRI) for assessing the coherence of blood oxygen level‐dependent (BOLD) fMRI timeseries among contiguous brain regions. ReHo serves as a proxy of oxygen consumption and captures similar aspects of brain function as fluorodeoxyglucose positron emission tomography (FDG‐PET). However, the clinical potential of ReHo remains unexplored. This study aimed to evaluate the feasibility of predicting changes in amyloid deposition using ReHo measures in middle to late‐aged participants at the earliest Alzheimer's inception and compare this technique's performance with FDG‐PET.

**Method:**

We examined 330 cognitively unimpaired individuals (mean age=61.0, SD=4.7) with available CSF Aβ42/40, *p*‐tau181, rs‐fMRI, FDG‐PET and Aβ‐PET data (Table 1). Average follow‐up time was 3.3 years (SD=0.3). CSF Aβ42 and Aβ40 were assessed with the NeuroToolKit, a panel of exploratory robust prototype assays, while *p*‐tau181 was measured with the Elecsys® Phospho‐Tau (181P) CSF immunoassay (both Roche Diagnostics International Ltd., Switzerland). rs‐fMRI data was preprocessed using fMRIPrep and XCP‐D, and ReHo maps were subsequently extracted. Mean FDG and mean ReHo were computed across regions of the Landau and Centiloid masks, respectively. We examined associations of mean FDG and ReHo at baseline with amyloid load at baseline, as well as with changes in amyloid load (follow‐up minus baseline, divided by follow‐up period), as assessed with CSF Aβ42/40 and PET Centiloid. Statistical models were adjusted by age, sex, *APOE* status, years of education and *p*‐tau181.

**Result:**

Cross‐sectional analysis revealed that lower ReHo levels were associated with lower CSF Aβ42/40 levels (*p* = 0.042), but not with PET Centiloids. FDG levels showed no significant association with either CSF Aβ42/40 or PET Centiloids (Figure 1). Longitudinally, lower baseline FDG levels were associated with increased PET amyloid burden over time (*p* = 0.008), while higher baseline ReHo levels were linked to decreases in CSF Aβ42/40 levels (*p* = 0.021) (Figure 2).

**Conclusion:**

The findings indicate that baseline ReHo and FDG provide complementary insights into amyloid dynamics in the preclinical phase of Alzheimer's disease. Specifically, ReHo shows potential as a predictive biomarker for early changes in soluble Aβ changes, warranting further investigation into its clinical utility alongside FDG‐PET.